# Serum surfactant protein D as a predictive biomarker for the efficacy of pirfenidone in patients with idiopathic pulmonary fibrosis: a post-hoc analysis of the phase 3 trial in Japan

**DOI:** 10.1186/s12931-020-01582-y

**Published:** 2020-11-30

**Authors:** Kimiyuki Ikeda, Hirofumi Chiba, Hirotaka Nishikiori, Arata Azuma, Yasuhiro Kondoh, Takashi Ogura, Yoshio Taguchi, Masahito Ebina, Hiroki Sakaguchi, Shogo Miyazawa, Moritaka Suga, Yukihiko Sugiyama, Toshihiro Nukiwa, Shoji Kudoh, Hiroki Takahashi, Pirfenidone Clinical Study Group in Japan

**Affiliations:** 1 Department of Respiratory Medicine and Allergology, School of Medicine, Sapporo Medical University, South 1, West 16, Sapporo, 060-8543 Japan; 2 Nippon Medical School, Tokyo, Japan; 3 Tosei General Hospital, Seto, Japan; 4 Kanagawa Cardiovascular and Respiratory Center, Yokohama, Japan; 5 Tenri Hospital, Tenri, Japan; 6 Tohoku Medical and Pharmaceutical University, Sendai, Japan; 7 Shionogi & Co., Ltd, Osaka, Japan; 8 Saiseikai Kumamoto Hospital, Kumamoto, Japan; 9 Jichi Medical University, Shimotsuke, Japan; 10 Tohoku University, Sendai, Japan; 11 Japan Anti-Tuberculosis Association, Tokyo, Japan

**Keywords:** Idiopathic pulmonary fibrosis, Pirfenidone, Biomarker, Surfactant protein D

## Abstract

**Background:**

Idiopathic pulmonary fibrosis (IPF) is a progressive, fatal disorder with a variable disease course. The recent advancement of antifibrotic therapy has increased the need for reliable and specific biomarkers. This study aimed to assess alveolar epithelial biomarkers as predictors for the efficacy of the antifibrotic drug pirfenidone.

**Methods:**

We conducted a post-hoc analysis of the prospective, multicenter, randomized, placebo-controlled, phase 3 trial of pirfenidone in Japan (total, n = 267; pirfenidone, n = 163; placebo, n = 104). Logistic regression analysis was performed to extract parameters that predicted disease progression, defined by a ≥ 10% relative decline in vital capacity (VC) from baseline and/or death, at week 52. For assessment of serum surfactant protein (SP)-D, SP-A and Krebs von den Lungen (KL)-6, all patients were dichotomized by the median concentration of each biomarker at baseline to the high and low biomarker subgroups. Associations of these concentrations were examined with changes in VC at each time point from baseline up to week 52, along with progression-free survival (PFS). Additionally, the effect of pirfenidone treatment on serial longitudinal concentrations of these biomarkers were evaluated.

**Results:**

In the multivariate logistic regression analysis, body mass index (BMI), %VC and SP-D in the pirfenidone group, and BMI and %VC in the placebo group were indicated as predictors of disease progression. Pirfenidone treatment reduced the decline in VC with statistical significance in the low SP-D and low SP-A subgroups over most of the treatment period, and also prolonged PFS in the low SP-D and low KL-6 subgroups. Furthermore, SP-D levels over time course were reduced in the pirfenidone group from as early as week 8 until the 52-week treatment period compared with the placebo group.

**Conclusions:**

Serum SP-D was the most consistent biomarker for the efficacy of pirfenidone in the cohort trial of IPF. Serial measurements of SP-D might have a potential for application as a pharmacodynamic biomarker.

*Trial registration* The clinical trial was registered with the Japan Pharmaceutical Information Center (JAPIC) on September 13, 2005 (registration No. JapicCTI-050121; http://Clinicaltrials.jp)

## Background

Idiopathic pulmonary fibrosis (IPF) is a chronic, progressive disease of unknown cause [1]. The prognosis is poor, with a median survival time of 3–5 years from diagnosis [2, 3]. The pathology of the disease is characterized by excessive extracellular matrix (ECM) deposition and remodeling, which is thought to result from repeated epithelial injury and subsequent aberrant wound healing [4]. The heterogeneity in the etiology of IPF is likely to lead to variations in disease progression.

This unpredictable disease course and a lack of reliable biomarkers pose challenges for the clinical management of IPF. Therefore, there is an urgent need for biomarkers that can predict disease progression precisely and early in the clinical course, aid in treatment decisions, and reflect treatment response in individual patients. Furthermore, circulating blood biomarkers can reflect disease processes across the total lung tissue, which also help in the identification of specific biologic pathways for treatment. The PROFILE trial [5–7], a prospective, multicenter, observational cohort study for patients with IPF, performed a sequential evaluation of > 100 serum protein biomarkers, and consequently demonstrated that biomarkers of the alveolar epithelial dysfunction and ECM turnover were important in patients with IPF who were naive to antifibrotic treatments. The investigators identified serum biomarkers of epithelial dysfunction, namely surfactant protein (SP)-D, cancer antigen (CA) 19–9, and CA-125, that might be prognostic of disease progression and mortality in patients with IPF [6].

Pirfenidone is an effective antifibrotic drug confirmed by pivotal clinical trials for IPF, CAPACITY and ASCEND [8, 9]. The recent advancement of effective antifibrotic therapy has accelerated studies for the identification of high-performance biomarkers [10]. Neighbors et al. [11] assessed the prognostic, predictive, and pharmacodynamic properties of 12 protein biomarkers related to the pathogenesis and progression of IPF, using blood samples from patients in the pirfenidone trials, and consequently identified some biomarkers associated with prognosis, including CC-chemokine ligand (CCL) 18; however, they were unable to identify biomarkers which predicted the efficacy of pirfenidone on pulmonary function consistently or reflected an appreciable pharmacodynamic response to pirfenidone. Unfortunately, their study did not include biomarkers associated with epithelial dysfunction such as SP-D, CA19-9 and CA-125 shown in the PROFILE trial [6, 12].

SP-D, SP-A and Krebs von den Lungen (KL)-6 are mainly produced by alveolar epithelial cells and secreted into the alveolar space [13–16]. Some of these proteins leak into the circulating blood, with only low concentrations usually detected in the sera in states of health [15, 16]. Results from previous studies have suggested that the levels of these serum proteins rise substantially in patients with IPF, and these levels might be used to distinguish IPF from other interstitial lung diseases [15–21]. In addition, increased serum concentration of these biomarkers was associated with disease progression and mortality in patients with IPF [15–19, 22–25]. Thus, these biomarkers were referenced in the updated international guideline for the idiopathic interstitial pneumonias [26] and have been used widely in the clinical management of IPF in Japan [13, 27]. We recently discovered that serum SP-D is a potential prognostic biomarker in patients with IPF receiving pirfenidone in our Japanese cohort study [28]. However, this investigation was based on a retrospective analysis of a small, single-center cohort; hence, because of the lack of a placebo control, it was not possible to examine whether SP-D predicted the efficacy of pirfenidone.

To solve the issues remaining in the previous studies and validate the predictive property of the three lung-specific epithelial biomarkers, we sought to conduct a post-hoc analysis of the phase 3 trial of pirfenidone in Japan [29].

## Methods

### Study design and participants

Our phase 3 trial of pirfenidone was a prospective, double-blind, randomized, placebo-controlled trial conducted at 73 centers to determine the efficacy and safety of pirfenidone over 52 weeks, prior to the approval for the treatment of IPF in Japan in 2008 [29]. The efficacy and safety results were reviewed by the independent Data and Safety Monitoring Board (DSMB). Patient eligibility, demographic and clinical characteristics, as well as methods, have been described previously [29]. The diagnosis of IPF was in accordance with the American Thoracic Society (ATS)/European Respiratory Society (ERS) consensus statement in 2000 and the “Clinical diagnostic criteria for idiopathic interstitial pneumonia (IIP)” (4th edition) in Japan [30, 31]. Eligible patients were allocated into the following three groups: high dose (1800 mg/day), low dose (1200 mg/day), and placebo, at a ratio of 2:1:2, respectively. In this post-hoc analysis, the high and low dose groups were analyzed together as the pirfenidone group, for simplification.

### Serum sample collection and measurements

Peripheral blood samples were collected at weeks 0, 2, 4, 8, 12, 16, 28, 40 and 52. Serum SP-D, SP-A and KL-6 were measured by ELISA using commercial assay kits (SP-D ELISA, Yamasa, Tokyo, Japan; SP-A Test Kokusai-F, Sysmex, Kobe, Japan; ED046, Eizai, Tokyo, Japan, respectively).

### Identification of predictors of disease progression

The primary endpoint of the original study was the change in vital capacity (VC) at week 52 from baseline. In this post-hoc analysis, the associations between clinical parameters at baseline and disease progression at week 52 were examined in each treatment group. These parameters included patient characteristics, physiological measurements and serum biomarker concentrations. Disease progression was defined by a ≥ 10% relative decline in VC from baseline and/or death. When the VC data could not be obtained because of worsening of respiratory symptoms, including acute exacerbation, the case was also classified as disease progression.

### Predictive property

In these analyses, all patients were dichotomized by the median concentration of each biomarker at baseline to the high and low biomarker subgroups. The difference of changes in VC between the pirfenidone and placebo groups at each time point from baseline was investigated by the high and low biomarker subgroups. In addition, we examined the association between biomarker concentrations at baseline and disease progression at week 52, along with progression-free survival (PFS) time, defined as the time from baseline to disease progression.

### Pharmacodynamic property

The effect of pirfenidone on changes in biomarker concentrations at each time point from baseline was assessed using a serial longitudinal measurement of the biomarkers. Serial changes were expressed as relative changes from baseline. In addition, in order to determine whether early changes in biomarker concentrations could predict outcomes, the association of changes in biomarker concentrations at week 16 with disease progression at week 52 and PFS time was investigated. In these analyses, all patients were dichotomized by changes in biomarker concentrations at week 16 to the increase and stable (equal and decrease) biomarker subgroups.

### Statistical analysis

We performed logistic regression analysis to explore the association between clinical parameters at baseline and disease progression at week 52. In this analysis, all parameters were analyzed as continuous variables, except for gender, smoking history (nominal variable), and dyspnea scale (ordinal variable). Factors with a p-value of < 0.10 in the univariate analysis were used as candidates of prognostic factors in the multivariate analysis. We used Fisher’s exact test for the comparisons of the proportion of patients with disease progression at week 52 between the pirfenidone and placebo groups and between the increase and stable biomarker subgroups. In these analyses, patients with worsening respiratory status in which pulmonary function testing could not be conducted and/or who died were classified as disease progression, and other missing data of VC at week 52 were input by the last observation carried forward (LOCF). A mixed model for repeated measurements (MMRM) was applied to calculate the adjusted mean of changes in VC at each time point from baseline. The MMRM consisted of treatment group, time point, interaction between them, and baseline VC as explanatory variables. We plotted survival curves using the Kaplan–Meier method, and the comparison of PFS time between groups was performed using the log-rank test. Summary statistics were calculated for the relative change in biomarker concentrations at each time point, and the comparison between the pirfenidone and placebo groups was performed by Wilcoxon rank-sum test. p values < 0.05 were considered an indication of statistical significance. Because this study was an exploratory post-hoc analysis of a clinical trial, we did not adjust the multiplicity of statistical tests. All statistical analyses were performed with SAS software version 9.2 or higher (SAS Institute Inc., Cary, NC, USA).

## Results

### Identification of predictors of disease progression

The full analysis set of 267 patients were examined in this post-hoc analysis of the phase 3 trial of pirfenidone. The flow diagram was reported previously [29]. Baseline characteristics and clinical data are shown in Table 1, and the number of patients with and without disease progression at week 52 is shown in Additional file 1: Table S1. The result of logistic regression analysis to extract factors that predict disease progression at week 52 is shown in Table 2. In the univariate analysis of the pirfenidone group, body mass index (BMI), smoking history, alveolar–arterial oxygen difference (AaDO_2_), %VC and SP-D were extracted with p < 0.10. From the multivariate analysis, BMI, %VC and SP-D were indicated as prognostic factors. In the univariate analysis of the placebo group, BMI, lowest peripheral capillary oxygen saturation (SpO_2_) and %VC were extracted with p < 0.10. From the multivariate analysis, BMI and %VC were indicated as prognostic factors.

**Table 1:** Baseline characteristics and clinical data of the study population

Parameter	All patients	Pirfenidone	Placebo
	(n = 267)	(n = 163)	(n = 104)
Gender			
Male, n (%)	213 (79.8%)	132 (81.0%)	81 (77.9%)
Female, n (%)	54 (20.2%)	31 (19.0%)	23 (22.1%)
Age, year	64.8 (6.9)	64.9 (6.7)	64.7 (7.3)
	(n = 267)	(n = 163)	(n = 104)
BMI	24.3 (3.0)	24.1 (2.9)	24.6 (3.2)
	(n = 267)	(n = 163)	(n = 104)
Smoking			
Current and former, n (%)	212 (79.4%)	129 (79.1%)	83 (79.8%)
Never, n (%)	55 (20.6%)	34 (20.9%)	21 (20.2%)
Dyspnea scale (modified by Fletcher)			
1, n (%)	53 (19.9%)	33 (20.2%)	20 (19.2%)
2, n (%)	143 (53.6%)	80 (49.1%)	63 (60.6%)
3, n (%)	71 (26.6%)	50 (30.7%)	21 (20.2%)
4 and 5, n (%)	0 (0.0%)	0 (0.0%)	0 (0.0%)
PaO_2_, Torr	80.7 (9.6)	80.5 (9.6)	81.0 (9.5)
	(n = 265)	(n = 161)	(n = 104)
AaDO_2_, Torr	17.7 (10.3)	17.9 (10.7)	17.4 (9.7)
	(n = 265)	(n = 161)	(n = 104)
Lowest SpO_2_ during a 6MET, %	89.0 (2.2)	89.0 (2.3)	89.0 (2.0)
	(n = 266)	(n = 162)	(n = 104)
VC, L	2.44 (0.67)	2.41 (0.65)	2.47 (0.70)
	(n = 265)	(n = 161)	(n = 104)
%VC, %	77.8 (17.4)	76.9 (17.4)	79.1 (17.4)
	(n = 265)	(n = 161)	(n = 104)
%DLCO, %	53.6 (17.8)	52.6 (17.6)	55.2 (18.2)
	(n = 264)	(n = 161)	(n = 103)
Serum SP-D, ng/mL	238.8 (156.8)	231.6 (140.8)	250.1 (179.1)
	(n = 267)	(n = 163)	(n = 104)
Serum SP-A, ng/mL	93.2 (51.6)	92.5 (43.8)	94.3 (62.0)
	(n = 267)	(n = 163)	(n = 104)
Serum KL-6, U/mL	1317.0 (800.3)	1318.2 (803.1)	1315.0 (799.6)
	(n = 267)	(n = 163)	(n = 104)

Data are presented as n (%), or mean (SD)

*BMI* body mass index, *PaO*_*2*_ partial pressure of oxygen in arterial blood, *AaDO*_*2*_ alveolar–arterial oxygen difference, *SpO*_*2*_ peripheral capillary oxygen saturation, *6MET* 6-min steady-state exercise test, *VC* vital capacity, *DLCO* diffusion capacity of the lung for carbon monoxide, *SP* surfactant protein, *KL* Krebs von den Lungen

**Table 2:** Predictors of IPF disease progression at week 52

Univariate analysis				Multivariate analysis			
Parameter	Odds ratio	90% CI	p-value	Parameter	Odds ratio	95% CI	p-value
*(A) Pirfenidone group*							
Gender; male vs. female	0.529	0.260, 1.077	0.1407	Gender; male vs. female			
Age	0.968	0.927, 1.011	0.2223	Age			
BMI	0.822	0.734, 0.921	0.0047	BMI	0.849	0.723, 0.998	0.0469
Smoking; current and former vs. never	0.444	0.224, 0.880	0.0510	Smoking; current and former vs. never	0.831	0.305, 2.266	0.7172
Dyspnea scale (modified by Fletcher)	1.359	0.884, 2.089	0.2413	Dyspnea scale (modified by Fletcher)			
PaO_2_	1.016	0.984, 1.050	0.4188	PaO_2_			
AaDO_2_	0.968	0.940, 0.998	0.0806	AaDO_2_	0.969	0.932, 1.007	0.1069
Lowest SpO_2_ during a 6MET	1.011	0.888, 1.151	0.8881	Lowest SpO_2_ during a 6MET			
%VC	0.939	0.918, 0.960	< 0.0001	%VC	0.943	0.916, 0.971	< 0.0001
%DLCO	1.011	0.995, 1.029	0.2653	%DLCO			
Serum SP-D	1.004	1.002, 1.006	0.0059	Serum SP-D	1.003	1.000, 1.006	0.0278
Serum SP-A	0.997	0.989, 1.004	0.4486	Serum SP-A			
Serum KL-6	1.000	0.999, 1.000	0.2321	Serum KL-6			
*(B) Placebo group*							
Gender; male vs. female	0.700	0.317, 1.546	0.4589	Gender; male vs. female			
Age	0.995	0.950, 1.042	0.8616	Age			
BMI	0.791	0.690, 0.908	0.0053	BMI	0.833	0.704, 0.985	0.0323
Smoking; current and former vs. never	0.509	0.222, 1.166	0.1803	Smoking; current and former vs. never			
Dyspnea scale (modified by Fletcher)	1.187	0.696, 2.025	0.5979	Dyspnea scale (modified by Fletcher)			
PaO_2_	0.973	0.937, 1.011	0.2349	PaO_2_			
AaDO_2_	1.005	0.971, 1.041	0.8057	AaDO_2_			
Lowest SpO_2_ during a 6MET	0.839	0.708, 0.994	0.0889	Lowest SpO_2_ during a 6MET	0.908	0.723, 1.141	0.4084
%VC	0.949	0.925, 0.973	0.0006	%VC	0.957	0.927, 0.987	0.0049
%DLCO	0.981	0.962, 1.001	0.1139	%DLCO			
Serum SP-D	1.001	0.999, 1.002	0.6032	Serum SP-D			
Serum SP-A	1.001	0.996, 1.007	0.7249	Serum SP-A			
Serum KL-6	1.000	1.000, 1.000	0.9441	Serum KL-6			

The disease progression was defined by a > 10% relative decline in vital capacity from baseline and/or death. Logistic regression analysis

*IPF* idiopathic pulmonary fibrosis, *CI* confidence interval, *BMI* body mass index, *PaO*_*2*_ partial pressure of oxygen in arterial blood, *AaDO*_*2*_ alveolar–arterial oxygen difference, *SpO*_*2*_ peripheral capillary oxygen saturation, *6MET* 6-min steady-state exercise test, *VC* vital capacity, *DLCO* diffusion capacity of the lung for carbon monoxide, *SP* surfactant protein, *KL* Krebs von den Lungen

### Serum biomarker concentrations

**Fig. 1:**
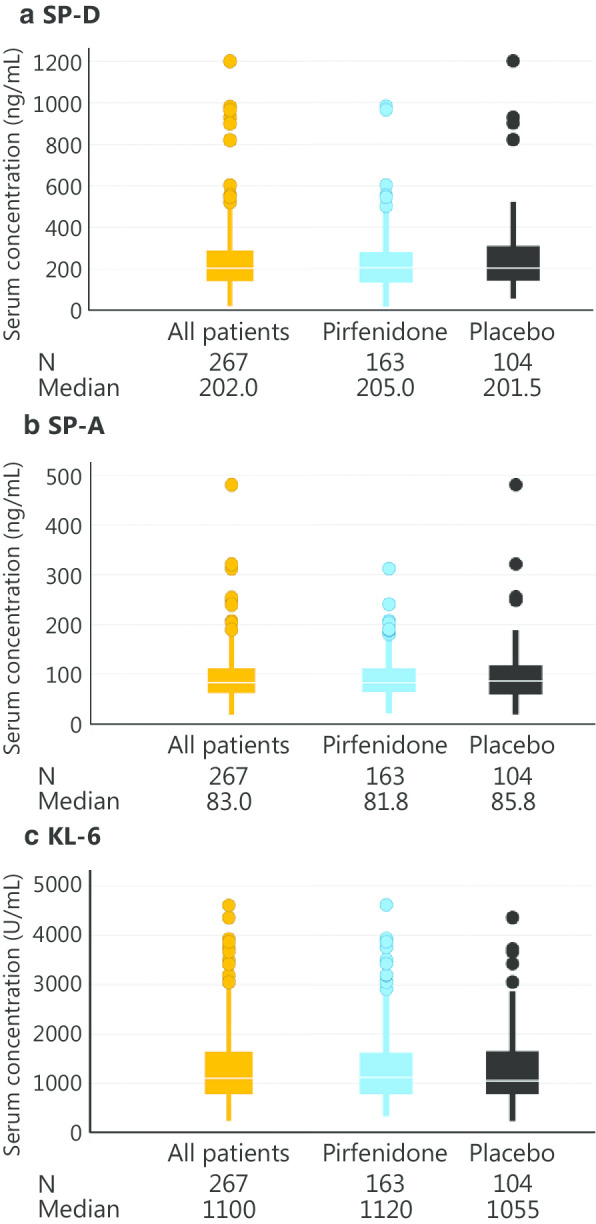
Distribution and median of serum concentration of **a** SP-D, **b** SP-A and **c** KL-6 at baseline in patients with IPF in the phase 3 trial of pirfenidone in Japan. No numerical difference in the distribution was observed between the pirfenidone and placebo groups for each biomarker

The distributions of serum concentrations of each biomarker at baseline are shown in Fig. 1. Median concentrations for all patients were as follows: SP-D 202.0 ng/mL (interquartile range [IQR], 138.5–287.0), SP-A 83.0 ng/mL (IQR, 60.9–112.0), and KL-6 1100 U/mL (IQR, 785–1635). There were no numerical differences in these biomarker concentrations between the pirfenidone and placebo groups.

### Predictive property

In the original trial, pirfenidone significantly reduced the decline in VC at week 52 and prolonged the PFS time, compared with placebo [29].

**Fig. 2:**
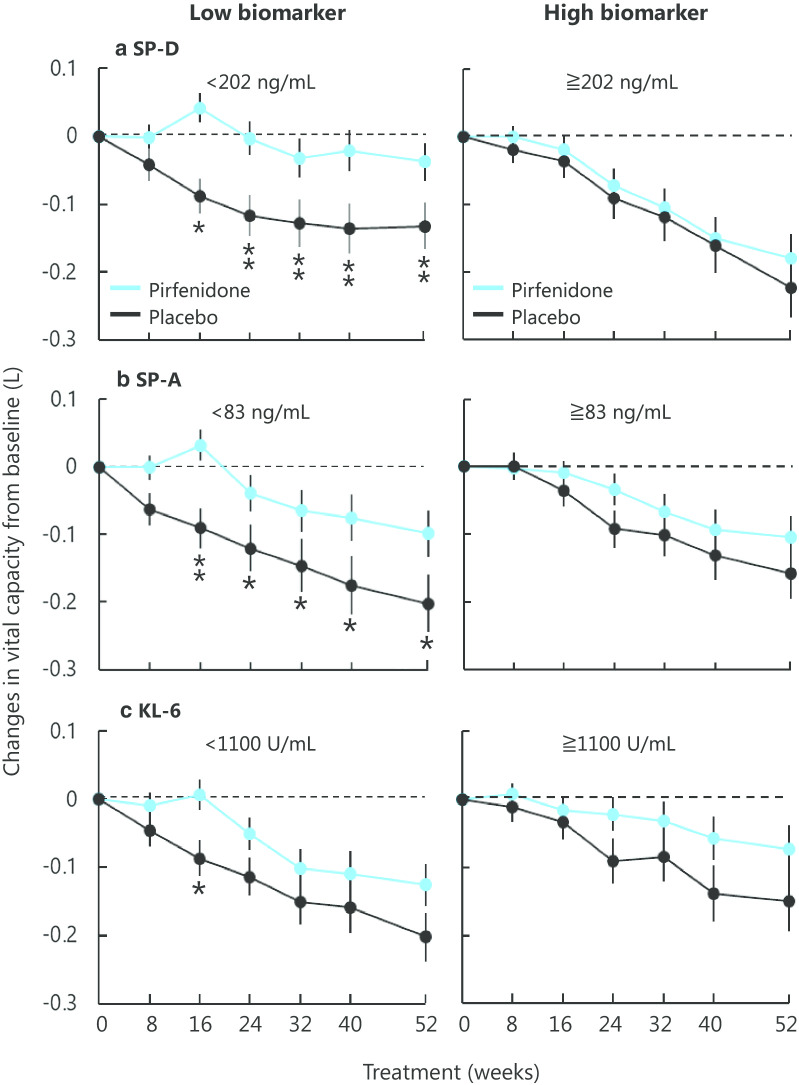
Relationship between serum concentrations of biomarkers at baseline and the efficacy of pirfenidone on changes in vital capacity from baseline. All patients were dichotomized by the median concentration of **a** SP-D, **b** SP-A and **c** KL-6 at baseline to the high and low biomarker subgroups. Blue lines, pirfenidone; black lines, placebo. Mean ± SE. Mean changes were calculated with the mixed model for repeated measurement (MMRM). *p < 0.05, **p < 0.01

The transition of VC over 52 weeks in the pirfenidone and placebo groups by the high and low biomarker subgroups is shown in Fig. 2. Pirfenidone treatment reduced the decline in VC compared with placebo in the low SP-D subgroup; the statistically significant difference was observed from as early as week 16 and continued until week 52, while the high SP-D subgroup showed little difference from placebo. Similar to SP-D, a statistically significant difference was observed between the pirfenidone and placebo groups in the low, but not high, SP-A subgroup from week 16 to 52. In contrast, the high and low KL-6 subgroups did not show any difference.

Table 3 shows the relationship between biomarker concentrations at baseline and disease progression at week 52. Pirfenidone treatment tended to associate with decreased risk of disease progression, compared to placebo in the low SP-D subgroup (p = 0.0949). Consistent results were also obtained using a marginal decline in VC, defined by a ≥ 5% relative decline, in an attempt to achieve more sensitive detection of disease progression (Additional file 1: Table S2) [32].

**Table 3:** Relationship between serum concentration of biomarkers at baseline and disease progression at week 52

Biomarker	Concentration at baseline	Treatment	Disease progression at week 52		p-value
			Yes	No	
SP-D	Low	Pirfenidone	15 (19.2%)	63 (80.8%)	0.0949
		Placebo	17 (33.3%)	34 (66.7%)	
	High	Pirfenidone	26 (32.5%)	54 (67.5%)	0.3605
		Placebo	21 (40.4%)	31 (59.6%)	
SP-A	Low	Pirfenidone	23 (28.0%)	59 (72.0%)	0.4394
		Placebo	17 (34.7%)	32 (65.3%)	
	High	Pirfenidone	18 (23.7%)	58 (76.3%)	0.0807
		Placebo	21 (38.9%)	33 (61.1%)	
KL-6	Low	Pirfenidone	19 (26.0%)	54 (74.0%)	0.2430
		Placebo	20 (37.0%)	34 (63.0%)	
	High	Pirfenidone	22 (25.9%)	63 (74.1%)	0.2397
		Placebo	18 (36.7%)	31 (63.3%)	

All patients were dichotomized by the median concentration of each biomarker at baseline to the high and low biomarker subgroups. The disease progression was defined by a > 10% relative decline in vital capacity from baseline and/or death. Fisher's exact test

*SP* surfactant protein, *KL* Krebs von den Lungen

**Fig. 3:**
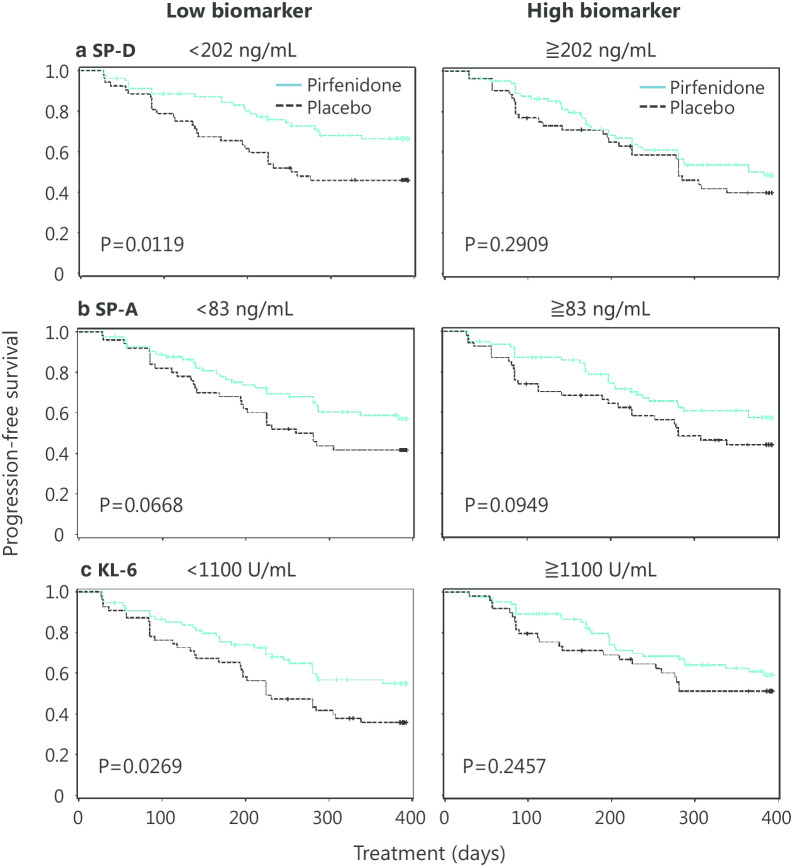
Relationship between serum concentrations of biomarkers at baseline and the efficacy of pirfenidone on progression-free survival. All patients were dichotomized by the median concentration of **a** SP-D, **b** SP-A and **c** KL-6 at baseline to the high and low biomarker subgroups. Blue lines, pirfenidone; black lines, placebo. The disease progression was defined by a > 10% relative decline in vital capacity from baseline and/or death. Progression-free survival was estimated using the Kaplan–Meier method and compared using the log-rank test

Figure 3 shows Kaplan–Meier PFS plots created by dichotomizing the patients according to the median concentration of each biomarker at baseline. In the low SP-D subgroup and the low KL-6 subgroup, pirfenidone prolonged PFS.

### Pharmacodynamic property

**Fig. 4:**
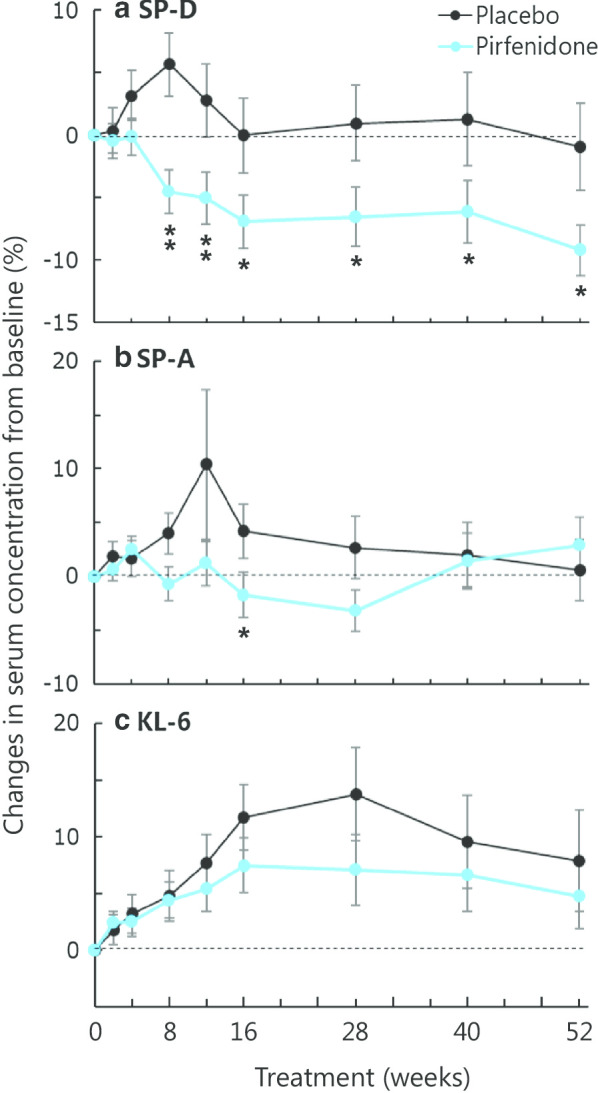
Effect of pirfenidone on relative changes in serum concentrations of **a** SP-D, **b** SP-A and **c** KL-6 from baseline. Blue lines, pirfenidone; black lines, placebo. Mean ± SE. The comparison between the pirfenidone and placebo groups was performed using the Wilcoxon rank-sum test: *p < 0.05, **p < 0.01

Figure 4 shows the transition of serum biomarker levels in the pirfenidone and placebo groups. The SP-D level was statistically significantly decreased in the pirfenidone group from week 8 to 52, although the level changed little over time in the placebo group. The SP-A level did not change much over time, and the KL-6 level was numerically elevated in both groups. The levels of SP-A and KL-6 in the pirfenidone group were not significantly different from those in the placebo group.

Conversely, changes in these three biomarker concentrations at week 16 were not associated with the risk of disease progression at week 52 in both the pirfenidone and placebo groups (Additional file 1: Table S3). Similarly, no statistical significance was observed for the association of changes in biomarker concentrations at week 16 with PFS time in both the pirfenidone and placebo groups, except KL-6 in the placebo group (Additional file 2: Fig. S1).

## Discussion

In our present post-hoc analysis of the phase 3 trial of pirfenidone in Japan, we used serum samples from our study patients to assess the predictive and pharmacodynamic properties of the three protein biomarkers related to progression of IPF. As a result, we found that single-point measurement of serum SP-D provided valuable information in estimating the efficacy of pirfenidone therapy (predictive biomarker). To our knowledge, this is the first multicenter large-scale study to show that the measurement of serum protein could predict the efficacy of antifibrotic therapy as measured by VC. Furthermore, based on the result shown in Fig. 4, serial longitudinal measurement of serum SP-D might address the crucial unmet need in the field of IPF research regarding the identification of specific molecular pathways for treatment (pharmacodynamic biomarker).

Our multivariate analysis revealed that SP-D was indicated as a predictive biomarker, independent of other clinical parameters, including respiratory physiological evaluation. Previous sub-nalyses of this clinical trial indicated the benefit of pirfenidone for patients evaluated as mild grade based on respiratory physiology [33, 34]. The independence of SP-D in the multivariate analysis suggested that biomarkers reflecting pathobiology are important, along with respiratory physiology. Next, we attempted to evaluate the association between biomarker concentrations and various outcome indicators in IPF. Table 3 and Additional file 2: Table S2 analyze the binary response of the presence or absence of disease progression at week 52 as determined using LOCF. Figure 2 analyze the continuous response of changes in VC over time using MMRM, and Fig. 3 analyze the time-to-event response of PFS using the Kaplan–Meier method. The results indicated that serum SP-D was the most consistent single baseline biomarker for the prediction of the efficacy of pirfenidone. On the other hand, SP-A and KL-6 showed some inconsistency between the different methods of analysis, leaving a challenge for robustness.

In a recent study by Neighbors et al. [11], the efficacy of pirfenidone was consistent regardless of baseline biomarker concentrations, and pirfenidone treatment had no meaningful pharmacodynamic effect on the serum levels of the prespecified biomarkers. On the other hand, baseline SP-D evaluated in our present study predicted the efficacy of pirfenidone therapy. This result confirms our discovery with our previous study [28]. Increasing serum concentrations of SP-D could identify the phenotype of IPF characterized by alveolar epithelial dysfunction*,* and its degree might determine the success of pirfenidone therapy. Therefore, SP-D could be of use in clinical practice and in future trials to identify individuals who are more likely to benefit from pirfenidone therapy.

The serial measurements of SP-D demonstrated its pharmacodynamic response to pirfenidone in the present study. Serum concentrations of SP-D in the pirfenidone group had decreased from early in the treatment, whereas these remained relatively constant in the placebo group over 52 weeks. Thus, SP-D may be useful for observing changes in pathobiology caused by treatment with pirfenidone. Changes in circulating biomarker concentrations might predict the progression of fibrosis earlier than the apparent decline displayed by physiological parameters such as VC and diffusion capacity of the lung for carbon monoxide (DLCO), and thus might be of use in determining response to therapy in clinical practice [35–37]. However, the decrease in the concentrations of SP-D at week 16 in patients receiving pirfenidone was not associated with outcomes in our present study. These data appear to be inconsistent, but they suggest that improvements in epithelial function alone did not result in improved outcomes based on the change in VC. In IPF with heterogeneous and complex pathobiology, linking short-term changes in a single biomarker to predicting the efficacy of treatment remains a challenge. Multiple biomarker panels, including those of epithelial dysfunction, ECM turnover and immune dysregulation, need to be developed.

The elevation of the serum concentrations of SP-D, SP-A, and KL-6 in IPF could be attributable to their abundant production by regenerating type II alveolar epithelial cells, and/or to the enhanced permeability of the air–blood barrier. Because of its molecular weight and biochemical properties, SP-D has been shown to easily leak into the blood from the alveolar space, and it is considered to be a sensitive biomarker for vascular permeability, as compared with SP-A and KL-6 [38, 39]. In our cohort, pirfenidone reduced serum concentrations of SP-D. On the other hand, Ronan et al. [40] evaluated the impact of pirfenidone on potential biomarkers of disease progression in blood, bronchoalveolar lavage fluids (BALF) and lung tissue. They identified six key biomarkers in BALF, namely three angiogenesis cytokines, two anti-inflammatory cytokines, and SP-D, which seemed to increase significantly during the treatment of pirfenidone. These findings and our data suggest that SP-D secreted from the alveolar epithelial cells to the alveolar space might leak less into the circulating blood upon treatment with pirfenidone.

As shown in our present study, pirfenidone is likely to exert a strong clinical effect in the pathological condition in which the epithelial damage, reflected by serum SP-D, remains at a minimal change. To preserve VC in the disease course, it might be better to start pirfenidone when the integrity of the epithelium could still be retained. Measurement of SP-D could allow clinicians to inform patients about the critical importance for early treatment. We believe that alveolar epithelial biomarkers, including SP-D, have the potential to address an urgent unmet need in the management of IPF. Our findings support continued evaluation of epithelial biomarkers with predictive and pharmacodynamic potential in future trials of IPF. The INMARK trial [41], which is assessing the effect of nintedanib on biomarkers found in the PROFILE trial in patients with IPF, might provide further valuable insights into the utility of alternative biomarkers related to epithelial dysfunction, as well as ECM turnover.

Our study has some limitations. First, this study was a post-hoc and exploratory analysis of the clinical trial. Second, the cohort of the presented study was not representative of real-world patients with IPF because the clinical trial excluded very mild or severe cases from enrolment. Meanwhile, our previous study included very mild or severe patients in real-world clinical conditions [28]; therefore, it might complement this limitation. Third, the treatment effect measured by LOCF might be overestimated and biased in favor of the pirfenidone group, because there might be more or earlier dropouts in the pirfenidone group than in the placebo group due to adverse events. However, similar tendency to LOCF were retained even if patients with missing values of VC at week 52 were excluded (data not shown). Finally, our study was a regional investigation with only Japanese patients, and it could not be ruled out that racial and regional differences might affect the results. Thus, an additional validation might be required before any of these epithelial biomarkers are employed clinically in other regions.

## Conclusions

We have shown that serum SP-D reflecting alveolar epithelial dysfunction in IPF is an informative biomarker for the efficacy of pirfenidone in patients with IPF. Serial measurements of epithelial biomarkers over treatment course had the potential to be used as pharmacodynamic and biologic indicators; however, it is challenging to estimate future changes in disease severity by short-term changes in biomarkers. Further validation of these findings in future prospective studies is warranted.

## Supplementary information

**Additional file 1:** Tables S1, S2 and S3.

**Additional file 2:** Fig. S1.

**Additional file 3:** Other contributions.

## Data Availability

The datasets used and analyzed during the current study are available from the corresponding author on reasonable request.

## Abbreviations

BMI: Body mass index
ECM: Extracellular matrix
IPF: Idiopathic pulmonary fibrosis
KL: Krebs von den Lungen
PFS: Progression-free survival
SP: Surfactant protein
VC: Vital capacity

## References

[1] Raghu G, Rochwerg B, Zhang Y, Garcia CAC, Azuma A, Behr J (2015). An official ATS/ERS/JRS/ALAT clinical practice guideline: treatment of idiopathic pulmonary fibrosis: an update of the 2011 clinical practice guideline. Am J Respir Crit Care Med.

[2] Nathan SD, Shlobin OA, Weir N, Ahmad S, Kaldjob JM, Battle E (2011). Long-term course and prognosis of idiopathic pulmonary fibrosis in the new millennium. Chest.

[3] Natsuizaka M, Chiba H, Kuronuma K, Otsuka M, Kudo K, Mori M (2014). Epidemiologic survey of Japanese patients with idiopathic pulmonary fibrosis and investigation of ethnic differences. Am J Respir Crit Care Med.

[4] Wolters PJ, Collard HR, Jones KD (2014). Pathogenesis of idiopathic pulmonary fibrosis. Annu Rev Pathol Mech Dis.

[5] Jenkins RG, Simpson JK, Saini G, Bentley JH, Russell AM, Braybrooke R (2015). Longitudinal change in collagen degradation biomarkers in idiopathic pulmonary fibrosis: an analysis from the prospective, multicentre PROFILE study. Lancet Respir Med.

[6] Maher TM, Oballa E, Simpson JK, Porte J, Habgood A, Fahy WA (2017). An epithelial biomarker signature for idiopathic pulmonary fibrosis: an analysis from the multicentre PROFILE cohort study. Lancet Respir Med.

[7] Organ L, Duggan AM, Oballa E, Taggart S, Simpson J, Kangombe A (2018). Biomarkers of collagen synthesis predict progression in the PROFILE idiopathic pulmonary fibrosis cohort. Respir Res..

[8] Noble PW, Albera C, Bradford WZ, Costabel U, Glassberg MK, Kardatzke D (2011). Pirfenidone in patients with idiopathic pulmonary fibrosis (CAPACITY): Two randomised trials. Lancet.

[9] King TE, Bradford WZ, Castro-Bernardini S, Fagan EA, Glaspole I, Glassberg MK (2014). A phase 3 trial of pirfenidone in patients with idiopathic pulmonary fibrosis. N Engl J Med.

[10] Somogyi V, Chaudhuri N, Torrisi SE, Kahn N, Müller V, Kreuter M (2019). The therapy of idiopathic pulmonary fibrosis: What is next?. Eur Respir Rev.

[11] Neighbors M, Cabanski CR, Ramalingam TR, Sheng XR, Tew GW, Gu C (2018). Prognostic and predictive biomarkers for patients with idiopathic pulmonary fibrosis treated with pirfenidone: post-hoc assessment of the CAPACITY and ASCEND trials. Lancet Respir Med.

[12] Kreuter M, Maher T (2018). Gazing into the crystal ball: can treatment response be predicted in IPF?. Lancet Respir Med.

[13] Walker SR, Williams MC, Benson B (1986). Immunocytochemical localization of the major surfactant apoproteins in type II cells, Clara cells, and alveolar macrophages of rat lung. J Histochem Cytochem.

[14] Voorhout WF, Veenendaal T, Kuroki Y, Ogasawara Y, van Golde LM, Geuze HJ (1992). Immunocytochemical localization of surfactant protein D (SP-D) in type II cells, Clara cells, and alveolar macrophages of rat lung. J Histochem Cytochem.

[15] Chiba H, Otsuka M, Takahashi H (2018). Significance of molecular biomarkers in idiopathic pulmonary fibrosis: a mini review. Respir Investig.

[16] Vij R, Noth I (2012). Peripheral blood biomarkers in idiopathic pulmonary fibrosis. Transl Res.

[17] Takahashi H, Fujishima T, Koba H, Murakami S, Kurokawa K, Shibuya Y (2000). Serum surfactant proteins A and D as prognostic factors in idiopathic pulmonary fibrosis and their relationship to disease extent. Am J Respir Crit Care Med.

[18] Greene KE, King JE, Kuroki Y, Bucher-Bartelson B, Hunninghake GW, Newman LS (2002). Serum surfactant proteins-A and -D as biomarkers in idiopathic pulmonary fibrosis. Eur Respir J.

[19] Wang K, Ju Q, Cao J, Tang W, Zhang J (2017). Impact of serum SP-A and SP-D levels on comparison and prognosis of idiopathic pulmonary fibrosis. Med (United States).

[20] White ES, Xia M, Murray S, Dyal R, Flaherty CM, Flaherty KR (2016). Plasma surfactant protein-D, matrix metalloproteinase-7, and osteopontin index distinguishes idiopathic pulmonary fibrosis from other idiopathic interstitial pneumonias. Am J Respir Crit Care Med.

[21] Raghu G, Richeldi L, Jagerschmidt A, Martin V, Subramaniam A, Ozoux ML (2018). Idiopathic pulmonary fibrosis: prospective, case-controlled study of natural history and circulating biomarkers. Chest.

[22] Takahashi H, Shiratori M, Kanai A, Chiba H, Kuroki Y, Abe S (2006). Monitoring markers of disease activity for interstitial lung diseases with serum surfactant proteins A and D. Respirology.

[23] Kinder BW, Brown KK, McCormack FX, Ix JH, Kervitsky A, Schwarz MI (2009). Serum surfactant protein-A is a strong predictor of early mortality in idiopathic pulmonary fibrosis. Chest.

[24] Song JW, Do KH, Jang SJ, Colby TV, Han S, Kim DS (2013). Blood biomarkers MMP-7 and SP-A: Predictors of outcome in idiopathic pulmonary fibrosis. Chest.

[25] Yokoyama A, Kondo K, Nakajima M, Matsushima T, Takahashi T, Nishimura M (2006). Prognostic value of circulating KL-6 in idiopathic pulmonary fibrosis. Respirology.

[26] Travis WD, Costabel U, Hansell DM, King TE, Lynch DA, Nicholson AG (2013). An official American Thoracic Society/European Respiratory Society statement: update of the international multidisciplinary classification of the idiopathic interstitial pneumonias. Am J Respir Crit Care Med.

[27] Homma S, Bando M, Azuma A, Sakamoto S, Sugino K, Ishii Y (2018). Japanese guideline for the treatment of idiopathic pulmonary fibrosis. Respir Investig.

[28] Ikeda K, Shiratori M, Chiba H, Nishikiori H, Yokoo K, Saito A (2017). Serum surfactant protein D predicts the outcome of patients with idiopathic pulmonary fibrosis treated with pirfenidone. Respir Med.

[29] Taniguchi H, Ebina M, Kondoh Y, Ogura T, Azuma A, Suga M (2010). Pirfenidone in idiopathic pulmonary fibrosis. Eur Respir J.

[30] American Thoracic Society (2000). Idiopathic pulmonary fibrosis: diagnosis and treatment: international consensus statement: American Thoracic Society (ATS), and the European Respiratory Society (ERS). Am J Respir Crit Care Med.

[31] Clinical diagnostic and treatment guidance for idiopathic interstitial pneumonias. In Nankodo Edited by: Japanese Respiratory Society’s Committee formulating diagnosis and treatment guideline for diffuse lung diseases. Tokyo. 2004;63–5. [in Japanese].

[32] Taniguchi H, Kondoh Y, Ebina M, Azuma A, Ogura T, Taguchi Y (2011). The clinical significance of 5% change in vital capacity in patients with idiopathic pulmonary fibrosis: Extended analysis of the pirfenidone trial. Respir Res.

[33] Azuma A, Taguchi Y, Ogura T, Ebina M, Taniguchi H, Kondoh Y (2011). Exploratory analysis of a phase III trial of pirfenidone identifies a subpopulation of patients with idiopathic pulmonary fibrosis as benefiting from treatment. Respir Res.

[34] Taguchi Y, Ebina M, Hashimoto S, Ogura T, Azuma A, Taniguchi H (2015). Efficacy of pirfenidone and disease severity of idiopathic pulmonary fibrosis: Extended analysis of phase III trial in Japan. Respir Investig.

[35] Yoshikawa T, Otsuka M, Chiba H, Ikeda K, Mori Y, Umeda Y (2020). Surfactant protein A as a biomarker of outcomes of anti-fibrotic drug therapy in patients with idiopathic pulmonary fibrosis. BMC Pulm Med.

[36] Wakamatsu K, Nagata N, Kumazoe H, Oda K, Ishimoto H, Yoshimi M (2017). Prognostic value of serial serum KL-6 measurements in patients with idiopathic pulmonary fibrosis. Respir Investig.

[37] Okuda R, Hagiwara E, Baba T, Kitamura H, Kato T, Ogura T (2013). Safety and efficacy of pirfenidone in idiopathic pulmonary fibrosis in clinical practice. Respir Med.

[38] Nishikiori H, Chiba H, Ariki S, Kuronuma K, Otsuka M, Shiratori M (2014). Distinct compartmentalization of SP-A and SP-D in the vasculature and lungs of patients with idiopathic pulmonary fibrosis. BMC Pulm Med.

[39] Shigemura M, Nasuhara Y, Konno S, Shimizu C, Matsuno K, Yamguchi E (2012). Effects of molecular structural variants on serum Krebs von den Lungen-6 levels in sarcoidosis. J Transl Med.

[40] Ronan N, Bennett DM, Khan KA, McCarthy Y, Dahly D, Bourke L (2018). Tissue and bronchoalveolar lavage biomarkers in idiopathic pulmonary fibrosis patients on pirfenidone. Lung.

[41] Maher TM, Stowasser S, Nishioka Y, White ES, Cottin V, Noth I (2019). Biomarkers of extracellular matrix turnover in patients with idiopathic pulmonary fibrosis given nintedanib (INMARK study): a randomised, placebo-controlled study. Lancet Respir Med.

