# Supplementary material for: Serum surfactant protein D as a predictive biomarker for the efficacy of pirfenidone in patients with idiopathic pulmonary fibrosis: a post-hoc analysis of the phase 3 trial in Japan
Source: Respir Res. 2020 Nov 30;21:316. doi: 10.1186/s12931-020-01582-y (PMC7706186; doi:10.1186/s12931-020-01582-y)
Supplement: Supplementary file 1 — Additional file 1: Tables S1, S2 and S3. [file 12931_2020_1582_MOESM1_ESM.docx]

**Table S1: Number of patients with and without disease progression at week 52.**

| Disease progression at week 52 | All patients  (n = 261) | Pirfenidone  (n = 158) | Placebo  (n = 103) |
| --- | --- | --- | --- |
| Yes, n (%) | 79 (30.3%) | 41 (25.9%) | 38 (36.9%) |
| No, n (%) | 182 (69.7%) | 117 (74.1%) | 65 (63.1%) |

According to the design of the original clinical trial, the last observation carried forward (LOCF) imputation was performed using the data after week 4. The following six patients in which the data of change in VC after applying LOCF was missing were excluded: VC values ​​at baseline were missing, two patients (two in the pirfenidone group); LOCF imputation was not performed because the patient dropped out by week 4, four patients (three in the pirfenidone group, one in the placebo group). The reasons for the dropout of these four patients were discontinuation due to adverse events (three in the pirfenidone group) and patient’s offer (one in the placebo group).

**Table S2: Relationship between serum concentration of biomarkers at baseline and disease progression (marginal decline in vital capacity) at week 52.**

| Biomarker | Concentration at baseline | Treatment | Disease progression at week 52 | | P-value |
| --- | --- | --- | --- | --- | --- |
|  |  |  | Yes | No |  |
| SP-D | Low | Pirfenidone | 28 (35.9%) | 50 (64.1%) | 0.0068 |
|  |  | Placebo | 31 (60.8%) | 20 (39.2%) |  |
|  | High | Pirfenidone | 38 (47.5%) | 42 (52.5%) | 0.0505 |
|  |  | Placebo | 34 (65.4%) | 18 (34.6%) |  |
| SP-A | Low | Pirfenidone | 33 (40.2%) | 49 (59.8%) | 0.0037 |
|  |  | Placebo | 33 (67.3%) | 16 (32.7%) |  |
|  | High | Pirfenidone | 33 (43.4%) | 43 (56.6%) | 0.1088 |
|  |  | Placebo | 32 (59.3%) | 22 (40.7%) |  |
| KL-6 | Low | Pirfenidone | 32 (43.8%) | 41 (56.2%) | 0.0123 |
|  |  | Placebo | 36 (66.7%) | 18 (33.3%) |  |
|  | High | Pirfenidone | 34 (40.0%) | 51 (60.0%) | 0.0476 |
|  |  | Placebo | 29 (59.2%) | 20 (40.8%) |  |

All patients were dichotomized by the median concentration of each biomarker at baseline to the high and low biomarker subgroups. The disease progression was defined by a >5% relative decline in vital capacity from baseline and/or death. Fisher's exact test.

Abbreviations: SP, surfactant protein; KL, Krebs von den Lungen.

**Table S3: Relationship between changes in serum concentration of biomarkers at week 16 and disease progression at week 52.**

| Biomarker | Treatment | Change in biomarker concentration at week 16 | Disease progression at week 52 | | P-value |
| --- | --- | --- | --- | --- | --- |
|  |  |  | Yes | No |  |
| SP-D | Pirfenidone | Stable | 39 (42.4%) | 53 (57.6%) | 0.8634 |
|  |  | Increase | 24 (44.4%) | 30 (55.6%) |  |
|  | Placebo | Stable | 34 (59.6%) | 23 (40.4%) | 0.5324 |
|  |  | Increase | 28 (66.7%) | 14 (33.3%) |  |
| SP-A | Pirfenidone | Stable | 36 (42.4%) | 49 (57.6%) | 0.8663 |
|  |  | Increase | 27 (44.3%) | 34 (55.7%) |  |
|  | Placebo | Stable | 26 (54.2%) | 22 (45.8%) | 0.1013 |
|  |  | Increase | 36 (70.6%) | 15 (29.4%) |  |
| KL-6 | Pirfenidone | Stable | 25 (39.1%) | 39 (60.9%) | 0.4040 |
|  |  | Increase | 38 (46.3%) | 44 (53.7%) |  |
|  | Placebo | Stable | 21 (58.3%) | 15 (41.7%) | 0.5244 |
|  |  | Increase | 41 (65.1%) | 22 (34.9%) |  |

All patients were dichotomized by changes in biomarker concentrations at week 16 from baseline to the increase and stable (equal and decrease) biomarker subgroups. The disease progression was defined by a >10% relative decline in vital capacity from baseline and/or death. Fisher's exact test.

Abbreviations: SP, surfactant protein; KL, Krebs von den Lungen.
