# Supplementary material for: Serum surfactant protein D as a predictive biomarker for the efficacy of pirfenidone in patients with idiopathic pulmonary fibrosis: a post-hoc analysis of the phase 3 trial in Japan
Source: Respir Res. 2020 Nov 30;21:316. doi: 10.1186/s12931-020-01582-y (PMC7706186; doi:10.1186/s12931-020-01582-y)
Supplement: Supplementary file 2 — Additional file 2: Fig. S1. [file 12931_2020_1582_MOESM2_ESM.pptx]

## Slide 1
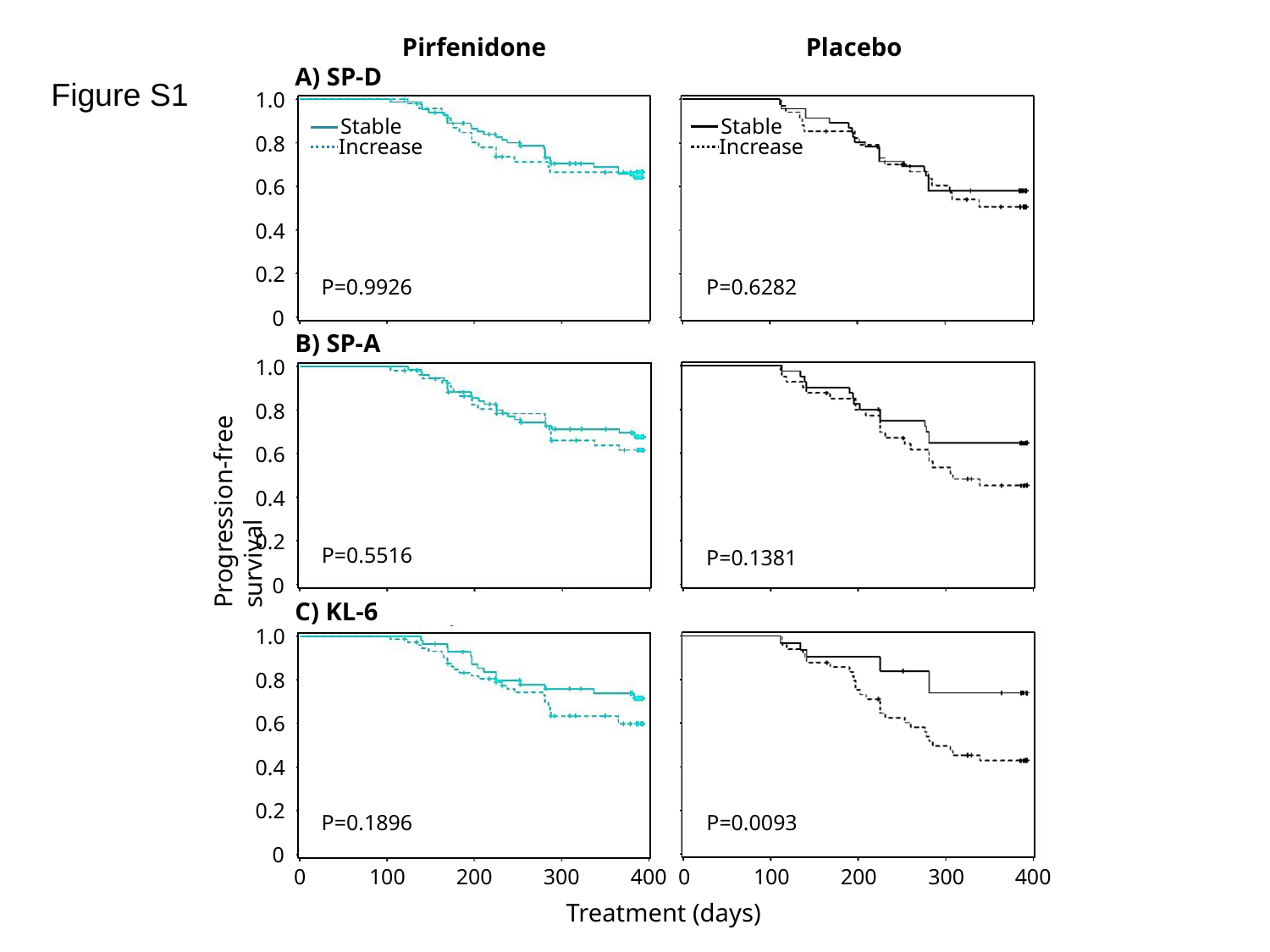

Pirfenidone
Placebo
A) SP-D
Figure S1
1.0
0.8
0.6
0.4
0.2
P=0.6282
P=0.9926
Stable
Increase
Stable
Increase
0
Progression-free survival
B) SP-A
1.0
0.8
0.6
0.4
0.2
P=0.5516
P=0.1381
0
C) KL-6
1.0
0.8
0.6
0.4
0.2
P=0.1896
P=0.0093
0
0
100
200
300
400
0
100
200
300
400
Treatment (days)

## Slide 2
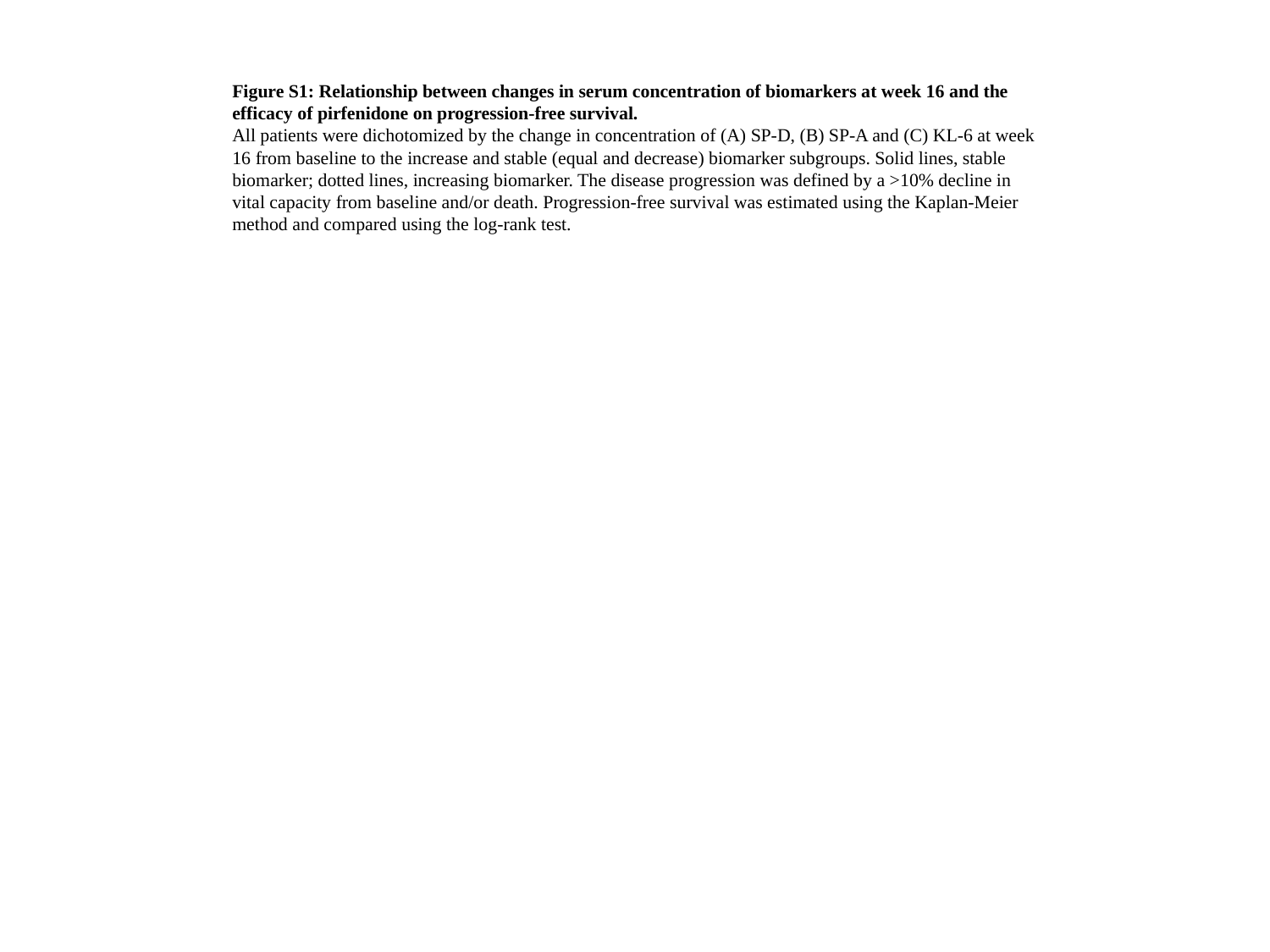

Figure S1: Relationship between changes in serum concentration of biomarkers at week 16 and the efficacy of pirfenidone on progression-free survival.
All patients were dichotomized by the change in concentration of (A) SP-D, (B) SP-A and (C) KL-6 at week 16 from baseline to the increase and stable (equal and decrease) biomarker subgroups. Solid lines, stable biomarker; dotted lines, increasing biomarker. The disease progression was defined by a >10% decline in vital capacity from baseline and/or death. Progression-free survival was estimated using the Kaplan-Meier method and compared using the log-rank test.
