# Supplementary material for: Serum surfactant protein D as a predictive biomarker for the efficacy of pirfenidone in patients with idiopathic pulmonary fibrosis: a post-hoc analysis of the phase 3 trial in Japan
Source: Respir Res. 2020 Nov 30;21:316. doi: 10.1186/s12931-020-01582-y (PMC7706186; doi:10.1186/s12931-020-01582-y)
Supplement: Supplementary file 3 — Additional file 3: Other contributions. [file 12931_2020_1582_MOESM3_ESM.docx]

**Other contributions**

The authors would like to thank M. Ando (Omotesando Yoshida Hospital, Kumamoto, Japan), S. Kitamura (Minami-Tochigi Hospital, Oyama, Tochigi, Japan), Y. Nakai (Tanpopo Clinic, Sendai, Miyagi, Japan), M. Takeuchi (Kitasato University, Tokyo, Japan) and A. Kondo (Niigata Tetsudo Kenshin Center, Niigata, Japan) of the independent Data and Safety Monitoring Board; K. Murata (Shiga University of Medical Science Hospital, Ohtsu, Shiga, Japan), M. Takahashi (Shiga University of Medical Science Hospital, Ohtsu, Shiga, Japan), H. Hayashi (Japanese Red Cross Okayama Hospital, Okayama, Japan), S. Noma (Tenri Hospital, Tenri, Japan), T. Johkoh (Osaka University Hospital, Osaka, Japan), H. Arakawa (Dokkyo Medical University Hospital, Shimotsuga, Tochigi, Japan) and K. Ichikado (Kumamoto University Hospital, Kumamoto, Japan) of the Imaging Central Judging Panel. The authors are also grateful to E. Tsuboi (Toranomon Hospital, Minato, Tokyo, Japan) for his expert advice on the 6-minute steady-state exercise test.

The members of Pirfenidone Clinical Study Group in Japan are as follows: T. Betsuyaku (Hokkaido University Hospital, Sapporo, Hokkaido), Y. Sugawara (Kyowakai Obihiro Respiratory Hospital, Obihiro, Hokkaido), S. Fujiuchi (Dohoku National Hospital, Asahikawa, Hokkaido), K. Yamauchi (Iwate Medical University Hospital, Morioka, Iwate), K. Konishi (Morioka Tsunagi Onsen Hospital, Morioka), M. Munakata (Fukushima Medical University Hospital, Fukushima), Y. Kimura (Tohoku University Hospital, Miyagi), Y. Ishii (Dokkyo Medical University Hospital, Shimotsuga, Tochigi), Y. Sugiyama (Jichi Medical University Hospital, Shimotsuga, Tochigi), K. Kudoh (International Medical Center of Japan, Shinjuku, Tokyo), T. Saito (Ibarakihigashi National Hospital, Naka, Ibaragi), T. Yamaguchi (JR Tokyo General Hospital, Shibuya, Tokyo), A. Mizoo (Tokyo Kosei Nenkin Hospital, Shinjuku), A. Nagai (Tokyo Women’s Medical University Hospital, Shinjuku), A. Ishizaka, K. Yamaguchi (Keio University Hospital, Shinjuku), K. Yoshimura (Toranomon Hospital, Minato, Tokyo), M. Oritsu (Japanese Red Cross Medical Center, Shibuya), Y. Fukuchi, K. Takahashi (Juntendo University Hospital, Bunkyo, Tokyo), K. Kimura (Toho University Omori Medical Center, Ota, Tokyo), Y. Yoshizawa (Tokyo Medical and Dental University Hospital, Bunkyo), T. Nagase (Tokyo University Hospital, Bunkyo), T. Hisada (Tokyo Teishin Hospital, Chiyoda, Tokyo), K. Ohta (Teikyo University Hospital, Itabashi, Tokyo), K. Yoshimori (Fukujuji Hospital, Kiyose, Tokyo), Y. Miyazawa, K. Tatsumi (Chiba University Hospital, Chiba), Y. Sasaki (Chiba-East Hospital, Chiba), M. Taniguchi (Sagamihara National Hospital, Sagamihara, Kanagawa), Y. Sugita (Saitama Cardiovascular and Respiratory Center, Kumagaya, Saitama), E. Suzuki (Niigata University Medical & Dental Hospital, Niigata), Y. Saito (Nishi-Niigata Chuo National Hospital, Niigata), H. Nakamura (Seirei Hamamatsu General Hospital, Hamamatsu, Shizuoka), K. Chida (Hamamatsu University School of Medicine, University Hospital, Hamamatsu), N. Kasamatsu (Hamamatsu Medical Center, Hamamatsu), H. Hayakawa (Tenryu Hospital, Hamamatsu), K. Yasuda (Iwata City Hospital, Iwata, Shizuoka), H. Suganuma (Shimada Municipal Hospital, Shimada, Shizuoka), H. Genma (Fukuroi Municipal Hospital, Fukuroi, Shizuoka), R. Tamura (Fujieda Municipal General Hospital, Fujieda, Shizuoka), T. Shirai (Fujinomiya City General Hospital, Fujinomiya, Shizuoka), J. Shindoh (Ogaki Municipal Hospital, Ogaki, Gifu), S. Sato (Nagoya City University Hospital, Nagoya, Aichi), O. Taguchi (Mie University Hospital, Tsu, Mie), Y. Sasaki (Kyoto Medical Center, Fushimi, Kyoto), H. Ibata (Mie Chuo Medical Center, Tsu, Mie), M. Yasui (Kanazawa University Hospital, Kanazawa, Ishikawa), Y. Nakano (Shiga Medical University Hospital, Otsu, Shiga), M. Ito, S. Kitada (Toneyama National Hospital, Toyonaka, Osaka), H. Kimura (Nara Medical University Hospital, Kashihara, Nara), Y. Inoue (Kinki-Chuo Chest Medical Center, Sakai, Osaka), H. Yasuba (Takatsuki Red Cross Hospital, Takatsuki, Osaka), Y. Mochizuki (Himeji Medical Center, Himeji, Hyogo), S. Horikawa, Y. Suzuki (Japanese Red Cross Wakayama Medical Center, Wakayama), N. Katakami (Institute of Biomedical Research and Innovation, Kobe, Hyogo), Y. Tanimoto (Okayama University Hospital, Okayama), Y. Hitsuda, N. Burioka (Tottori University Hospital, Yonago, Tottori), T. Sato (Okayama Medical Center, Okayama), N. Kohno, A. Yokoyama (Hiroshima University Hospital, Hiroshima), Y. Nishioka (Tokushima University Hospital, Tokushima), N. Ueda (Ehime Prefectural Central Hospital, Matsuyama, Ehime), K. Kuwano (Kyushu University Hospital, Fukuoka), K. Watanabe (Fukuoka University Hospital, Fukuoka), H. Aizawa (Kurume University Hospital, Kurume, Fukuoka), S. Kohno, H. Mukae (Nagasaki University Hospital of Medicine and Dentistry, Nagasaki), H. Kohrogi (Kumamoto University Hospital, Kumamoto), J. Kadota, I. Tokimatsu, E. Miyazaki (Oita University Hospital, Yufu, Oita), T. Sasaki (Miyazaki University Hospital, Miyazaki), M. Kawabata (Minami Kyushu National Hospital, Aira, Kagoshima).
